# Distribution of maternity waiting homes and their correlation with perinatal mortality and direct obstetric complication rates in Ethiopia

**DOI:** 10.1186/s12884-019-2356-x

**Published:** 2019-06-25

**Authors:** Gizachew Tadele Tiruneh, Yayeh Negash Getu, Mahbub Ali Abdukie, Geremew Gonfa Eba, Emily Keyes, Patricia E. Bailey

**Affiliations:** 1 The Last Ten Kilometers (L10K) Project, JSI Research & Training Institute, Inc., Addis Ababa, Ethiopia; 2 UNICEF, Addis Ababa, Ethiopia; 3 UNFPA, Addis Ababa, Ethiopia; 4 Ethiopian Public Health Institute, Addis Ababa, Ethiopia; 5 FHI 360, 359 Blackwell Street, Suite 200, Durham, NC 27701 USA; 6 Averting Maternal Death and Disability, Durham, NC USA

**Keywords:** Emergency obstetric and newborn care, Ethiopia, Obstetric complications, Maternity waiting homes, Perinatal death, Stillbirth

## Abstract

**Background:**

Ethiopia has been expanding maternity waiting homes to bridge geographical gaps between health facilities and communities in order to improve access to skilled care. In 2015, the Ministry of Health revised its national guidelines to standardize the rapid expansion of waiting homes. Little has been done to document their distribution, service availability and readiness. This paper addresses these gaps as well as their association with perinatal mortality and obstetric complication rates.

**Methods:**

We utilized data from the 2016 national Emergency Obstetric and Newborn Care assessment, a census of 3804 public and private health facilities. Data were collected between May and December 2016 through interviews with health care workers, record reviews, and observation of infrastructure. Descriptive statistics describe the distribution and characteristics of waiting homes and linear regression models examined the correlation between independent variables and institutional perinatal and peripartum outcomes.

**Results:**

Nationally, about half of facilities had a waiting home. More than two-thirds of facilities in Amhara and half of the facilities in SNNP and Oromia had a home while the region of Gambella had none. Highly urbanized regions had few homes.

Conditions were better among homes at hospitals than at health centers. Finished floors, electricity, water, toilets, and beds with mattresses were available at three (or more) out of four hospital homes. Waiting homes in pastoralist regions were often at a disadvantage.

Health facilities with waiting homes had similar or lower rates of perinatal death and direct obstetric complication rates than facilities without a home. The perinatal mortality was 47% lower in hospitals with a home than those without. Similarly, the direct obstetric complication rate was 49% lower at hospitals with a home compared to hospitals without.

**Conclusions:**

The findings should inform regional maternal and newborn improvement strategies, indicating gaps in the distribution and conditions, especially in the pastoralist regions. The impact of waiting homes on maternal and perinatal outcomes appear promising and as homes continue to expand, so should efforts to regularly monitor, refine and document their impact.

## Background

Approximately three-quarters of maternal and newborn deaths are clustered around the time of labor and delivery, indicating that timely access to quality intrapartum services is a key factor to maternal and newborn survival [1–4]. This presents a challenge in many low and middle-income countries (LMICs), where access to evidence-based life-saving interventions is often low [5, 6]. Distance to care, geographical barriers, lack of transport, lack of communication, associated costs, and reluctance to move within reach of care before labor are all common obstacles [4, 7–9].

Although many women overcome challenges to visit a health facility for antenatal care (ANC), it is not always possible for them to receive facility-based care throughout the continuum of pregnancy, delivery and postpartum period. In Ethiopia, where 84% of the population lives in rural areas [10], the coverage of postpartum and neonatal care services remains low. High “fall out” rates across the continuum of maternal and newborn care have been observed. The 2016 Ethiopian Demographic and Health Survey reports that while 62% of pregnant women made at least one skilled ANC visit, only 28% delivered their babies under the assistance of a skilled person, and even fewer (17%) received postnatal care within 48 h of the birth [11] despite institutional delivery levels showing a three-fold increase between 2011 and 2016 [11, 12].

In addition to providing routine ANC, intrapartum, and postnatal care, ensuring an equitable distribution of emergency obstetric and newborn care (EmONC) facilities and strengthening the referral system are critical strategies to reducing both maternal and newborn mortality in LMICs. Another key strategy involves assisting women to move closer to an EmONC facility before labor. This helps to ensure access to care should a pregnant woman or a newborn require it [4, 13]. Maternity waiting homes (MWHs)^1^ have been found to provide this opportunity. This is an alternative strategy to emergency referral as it avoids the “second delay,” or the time and costs incurred to transfer a woman in labor from home to appropriate definitive care [4, 14].

Ethiopia has implemented MWHs since 1976, using traditional cottages that were built by the people of the locality to bridge the geographical gaps between facilities and communities [15]. Their characteristics, typologies and their capacity for clients vary widely across regions [16]. To standardize the rapid national expansion of MWHs, in 2015 the Ethiopian Ministry of Health revised the MWH guidelines distributed to facilities [17]. Accordingly, the explicit goal was to increase access to skilled professionals at birth, especially at health centers [17]. Although the focus on health centers departs from the historical justification for MWHs to target women at high risk of adverse pregnancy outcomes or women who live in remote areas, this strategy was one of several that have driven the increased coverage of institutional delivery care in the last five years [18]. It also reflects WHO’s thinking: “Maternity waiting homes are recommended to be established close to a health facility, where essential childbirth care and/or care for obstetric and newborn complications is provided, to increase access to skilled care for populations living in remote areas or with limited access to services” [19].

Recent systematic and scoping reviews of observational studies examining the impact of MWHs on maternal and perinatal mortality demonstrated that their use is linked to the uptake of maternal health services and to a reduced risk of maternal and perinatal death in LMICs in general, and in Ethiopia in particular [14, 20]. Dadi et al. showed that in Ethiopia MWHs contributed to an 83% reduction in stillbirths and a 91% reduction in maternal deaths when compared to women who did not attend a MWH [14]. Evidence also showed that access barriers to and use of MWHs differed greatly among countries, as did service and management standards [14]. The same was true within regions in Ethiopia [16]. However, a wide knowledge gap remains regarding the outcomes of neonates born at MWHs [14, 20]. There is also little evidence to support the effectiveness of MWHs to reduce neonatal and maternal morbidity, such as the incidence of obstetric complications in LMIC settings [14, 21].

In Ethiopia, little has been done to document the distribution, service availability and readiness of MWHs. Similarly, a better understanding is needed of the impact of MWHs on improving maternal and newborn health outcomes. This paper assesses the availability and distribution of MWHs and their association with perinatal mortality and obstetric complication rates based on the findings of the 2016 national EmONC assessment for Ethiopia.

## Methods

### Study setting

#### Ethiopian health system

The country has a decentralized health system with three tiers where the first level provides primary health care and acts as the major platform for health service delivery. It consists of one primary hospital with four or five primary health care units (PHCUs). A PHCU is composed of a health center and five satellite health posts to serve approximately 25 thousand people. Health centers are staffed with health officers, nurses, midwives, and laboratory technicians to provide primarily preventive care including ANC, delivery and post-natal care, curative, inpatient and ambulatory services, including maternal and child health (MCH) services. It serves as a referral center and administrative and technical linkage to health posts. A primary hospital provides inpatient and ambulatory services to an average population of 100,000. It also provides emergency surgical services, including cesarean sections and access to blood transfusion services, and serves as a referral center for the health centers in its catchment area while serving as a practical training center for nurses and other paramedical health professionals. General hospitals provide care at the secondary level to a catchment population of approximately one million people. They serve as referral and training centers for primary hospitals and mid-level professionals. The third level is a specialized hospital that serves a catchment population of about five general hospitals or 5 million people.

All public health centers and hospitals, as well as private hospitals and MCH specialty clinics, are expected to provide delivery services.

#### National maternal and newborn health service initiatives

Ethiopia is committed to improving maternal and newborn health outcomes and its targets are aligned with those of the Sustainable Development Goals. To improve outcomes, Ethiopia aims to strengthen health systems to provide universal access to high quality promotive, preventive, curative, and rehabilitative services. This strategy is laid out in the Health Sector Transformation Plan (HSTP 2015–2020). During the HSTP period, the Federal Ministry of Health has developed different strategies and initiatives including the establishment of effective clinical mentorship and quality improvement initiatives. Moreover, the government seeks to improve access to and utilization of EmONC services by promoting facility delivery, expanding MWHs at health centers [17], strengthening referral linkages through the procurement and distribution of ambulances, expanding the number of health facilities, and the number of midwives, emergency surgical officers, and specialty professionals to ensure EmONC services [22]. Maternity waiting homes are expected to be available in most rural health centers which are closer to the rural population than other health facilities.

### Data and data collection

This is a secondary analysis of the 2016 national EmONC assessment [18], a national cross-sectional census of 3804 public and private health facilities that provided maternal and newborn health services. All public hospitals (referral, general, primary) and health centers, and all private (for-profit and not-for-profit) facilities (hospitals, MCH specialty centers, MCH specialty clinics, and higher clinics) that reported having attended births in the 12 months prior to the survey were included in the study. Facilities classified as medium clinics or below were excluded per the guidance of the Food, Medicine and Health Care Administration and Control Authority of Ethiopia, who sets out which facilities are expected to provide childbirth services.

The 2008 Ethiopia EmONC assessment modules (questionnaires) and a set of survey tools revised by Columbia University’s Averting Maternal Death and Disability Program (AMDD) in 2014 were adapted to the national context. The Ethiopian Public Health Institute (EPHI) designed an electronic data collection template using CSPro 6.1.

Data were collected between May and December 2016 through interviews with health care workers, record reviews, and observation of infrastructure. The overall assessment utilized 14 facility-based modules. Ethiopia was the first country to test the MWH module. It included data related to the infrastructure, support, and features of the MWH as reported by the facility medical director or designee.

### Analysis

For this secondary analysis, we used the facility case summary and maternity waiting home modules. Data were managed using CSPro 6.1 programming and exported to Stata 15.1 for statistical analysis [23]. Distribution, infrastructure, and characteristics of MWHs were described and the association between independent and dependent variables were analyzed using univariate and multivariate linear regression models where the unit of analysis was the facility.

### Variables and definitions

#### Dependent variables for regression models

The outcome variables considered in this analysis were the institutional perinatal death rate (PDR) and direct obstetric complication rate (DOCR) in the 12-month period preceding the assessment. We defined perinatal deaths as all stillbirths (macerated and fresh) and all live births who died within 24 h or before discharge, whichever came first. The perinatal deaths were divided by the number of deliveries that took place in the facility over the same period and multiplied by 100. The DOCR was defined as the proportion of admitted women who had a major obstetric complication (antepartum or postpartum hemorrhage, retained placenta, severe pre-eclampsia or eclampsia, severe abortion complications, uterine rupture, ectopic pregnancy and prolonged/obstructed labor) as well as any other direct obstetric complication (multiple gestation, premature rupture of membranes, etc.). It was calculated as the number of women with obstetric complications treated divided by the number of deliveries recorded in the same facility, multiplied by 100. We performed logarithmic transformations on each outcome variable prior to running the models to achieve a more normal distribution; thus, regression coefficients should be interpreted as percent change.

#### Independent variables for regression models

The independent variable of primary interest was the availability of a MWH. Moreover, we included region, managing authority of the facility, location of facility (urban/rural), availability of motor transport, density of skilled birth attendants (SBAs) per annual deliveries, and volume of annual deliveries. Other variables and their operational definitions used in this study are presented below.

*EmONC facility:* EmONC is defined as a set of life-saving interventions used to treat the major obstetric causes of morbidity and mortality. To assess the level of care, the performance of these signal functions in the last 3 months defines whether a facility is classified as providing basic EmONC (BEmONC) or comprehensive EmONC (CEmONC). BEmONC services comprise: 1) administration of parenteral antibiotics to prevent puerperal infection or treat abortion complications; 2) administration of parenteral anticonvulsants for treatment of eclampsia and preeclampsia; 3) administration of parenteral uterotonic drugs for postpartum hemorrhage; 4) manual removal of the placenta; 5) assisted vaginal delivery (vacuum extractions); 6) removal of retained products of conception; and 7) neonatal resuscitation with bag and mask. CEmONC services comprise cesarean sections and blood transfusions, in addition to all BEmONC functions [13].

*Index of MWH infrastructure and amenities:* an index score was calculated for each MWH, measured by 10 infrastructure and amenity indicators listed in Table 1. Each item was given a score of 0–2 points: 2 for each item available that met the standard, 1 for partial availability and 0 for not available or below the standard. Three items had a maximum of 1 point. Items were given equal weights and a total score was generated; maximum score being 17. Waiting homes that scored 13 or more were categorized as optimal, scores in the range of 9–12 points were ranked as basic, and scores less than 9 were considered substandard.

**Table 1:** Items used to measure MHW index score

Items	Scores		
	0 points	1 point	2 points
Infrastructure			
Electricity	No source	Any one source	Grid with backup
Water	None	Any water source	Water within 500 m
Toilet/latrine available	None	Any latrine	Private latrine for MWH patients
Finished floor material	Natural	Rudimentary	Finished
Patient-centeredness			
Sleeping arrangement	Not private (shared space)	Shared space with privacy curtain	Private sleeping space
Sleeping surface	Mat on the floor or other	Mattress on floor	Mattress on bed
Facility provides food	No	Yes	–
Has a garden	No	Yes	–
Welcomes families	No	Yes	–
Provides education	No	Last provided a week ago or before	Provided within last week

## Results

The study results are presented in three sections: 1) distribution of MWHs, 2) infrastructure and condition of MWHs, and 3) univariate and multivariate analyses of the relationship between the availability of MWHs, other facility characteristics and perinatal death rates (PDR) and direct obstetric complication rates (DOCR).

### Distribution of MWHs

Nationally, just over half of facilities that provide childbirth services had MWHs at the time of the assessment (Table 2). Availability of MWHs varied by region, facility type, and managing authority. More than two-thirds of facilities in Amhara region and half of the facilities in SNNP and Oromia had them. Gambella region had no facility with a MWH. Fewer than one in 10 facilities in the urban regions, namely Addis Ababa, Dire Dawa, and Harari, had a MWH.

**Table 2:** Percentage of facilities with a maternity waiting home according to type of infrastructure, by region, facility type, managing authority, and urban/rural location

	No. of facilities	Facilities with a MWH n (%)	Type of infrastructure n (%)	
			Stand-alone MWH	Room within facility
National	3804	2001 (52.6)	765 (20.1)	1236 (32.5)
Region				
Tigray	255	93 (36.5)	23 (9.0)	70 (27.5)
Afar	77	5 (6.5)	1 (1.3)	4 (5.2)
Amhara	876	631 (72.1)	210 (24.0)	421 (48.1)
Oromia	1405	792 (56.4)	326 (23.2)	466 (33.2)
Somali	161	11 (6.8)	5 (3.1)	6 (3.7)
Benishangul-Gumuz	43	11 (25.6)	0 (0.0)	11 (25.6)
SNNP	773	444 (57.5)	200 (25.9)	244 (31.6)
Gambella	27	0 (0.0)	0 (0.0)	0 (0.0)
Harari	15	1 (6.7)	0 (0.0)	1 (6.7)
Addis Ababa	151	11 (7.3)	1 (0.7)	10 (6.6)
Dire Dawa	21	2 (9.5)	0 (0.0)	2 (9.5)
Facility type				
Hospitals & MCH specialty centers	316	58 (18.3)	19 (6.0)	39 (12.3)
Health centers & clinics	3488	1943 (55.7)	746 (21.4)	1196 (34.3)
Managing authority				
Public	3662	1984 (54.2)	758 (20.7)	1196 (33.5)
Private, for-profit	83	0 (0.0)	0 (0.0)	0 (0.0)
Private, not-for-profit	59	17 (28.8)	7 (11.9)	10 (16.9)
Location				
Urban	1497	724 (48.4)	290 (19.4)	434 (29.0)
Rural	2307	1277 (55.4)	476 (20.6)	801 (34.7)

One-fifth of facilities had stand-alone MWHs, while a third maintained a MWH as an existing room in the facility.

### Infrastructure and characteristics of MWHs

Almost all MWHs received food stuff, furniture, construction of infrastructure, and maintenance support either from the community, faith-based organizations, non-governmental organizations, the facility itself, or from a combination of these sources (data not shown). More than 84% of MWHs provided food and/or health education to women during their stay. About two-thirds (65%) of MWHs had finished floors of vinyl, polished wood, cement, brick, or carpet. About three-quarters of MWHs had a latrine for occupants, and 73% had a source of electricity. Fifty-seven percent of MWHs had water for the women. Availability of these items varied by region, facility type, managing authority and location (data not shown).

Overall, in 45% of MWHs, women shared sleeping space and in 58% of MWHs women slept on beds with mattresses. Nationally, MWHs could accommodate a mean of 7.4 women at a given time. Regional analysis showed that the mean maximum capacity of women per MWH ranged from 12.0 in SNNP to 2.4 in Addis Ababa. Capacity of MWHs also varied by facility type (11.0 in hospitals vs 7.3 in health centers) and managing authority (7.4 in public vs 12.6 in private not-for-profit facilities). More than half (55%) of MWHs consisted of 1 room, 25% had 2 rooms and 20% 3 or more rooms (data not shown).

**Fig. 1:**
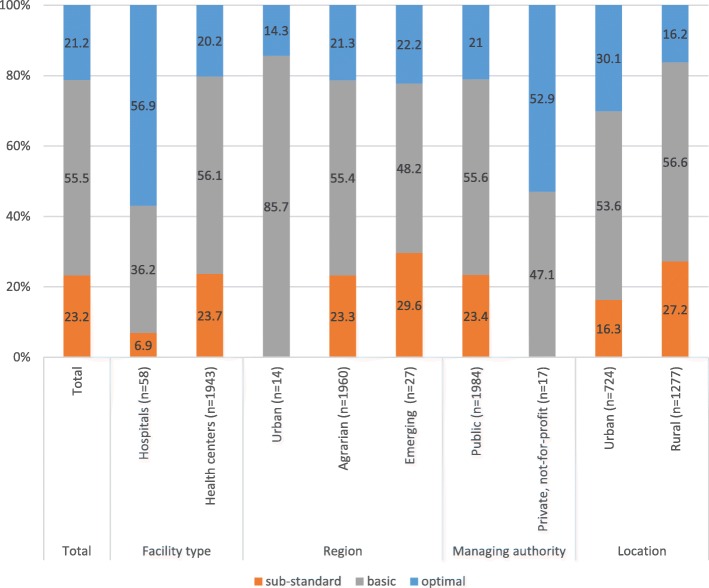
Percent distribution of facilities with maternity waiting homes according to summary index score (infrastructure and amenities), by facility characteristics

In summary, based on the infrastructure characteristics as well as patient-centered items given in Table 1, among the 2001 facilities with a MWH, about one-fifth had optimal infrastructure and amenities, 56% were considered basic and 23% were considered substandard (Fig. 1).

### Perinatal death rates (PDR) and direct obstetric complication rates (DOCR)

Health facilities with MWHs had similar or lower rates of both outcomes than facilities without a MWH (Table 3). At the national level, both the PDR and DOCR were about 4 times higher in hospitals than in health centers. Facilities with motorized transport had higher rates of perinatal death and obstetric complications than facilities without transport. The highest PDRs were seen in facilities in the pastoralist regions. As delivery volume increased at health centers, PDR decreased, but among hospitals, the highest volume facilities had the highest PDRs. On the other hand, hospitals and health centers with higher volumes of births had lower obstetric complication rates. Higher than average DOCRs were observed in urban regions, in private for-profit facilities, in facilities providing few signal functions, and those with the greatest number of skilled birth attendants (SBA) (per 100 deliveries).

**Table 3:** Average perinatal death rates and direct obstetric complication rates for selected health facility characteristics, by facility type

	Perinatal deaths per 100 deliveries				Direct complications per 100 deliveries			
	Health Centers		Hospitals		Health Centers		Hospitals	
	mean	n	mean	N	mean	N	mean	n
Total	1.1	3446	4.5	294	4.8	3446	19.2	294
Maternity waiting home								
No	1.1	1510	4.6	239	5.8	1510	21.1	239
Yes	1.1	1936	4.1	55	3.9	1936	10.9	55
Region								
Urban (Addis Ababa, Dire Dawa, Harari)	0.6	126	2.4	58	28.7	126	23.4	58
Agrarian (Amhara, Oromia, SNNP, Tigray)	1.0	3051	5.0	215	3.8	3051	18.1	215
Pastoralist (Gambella, Benishangul-Gumuz, Afar, Somali)	2.2	269	5.5	21	4.7	269	18.7	21
Managing authority								
Public	1.1	3384	5.3	218	4.7	3384	17.1	218
Private, for-profit	1.2	22	1.9	57	8.7	22	29.1	57
Private, not-for-profit	0.9	40	3.2	19	11.0	40	13.9	19
Location								
Urban	1.2	1199	4.5	271	8.1	1199	19.9	271
Rural	1.0	2247	4.6	23	3.0	2247	11.5	23
EmONC status								
Fully EmONC	1.3	185	5.6	179	7.8	185	18.2	179
Missing 1 or 2 SFs	1.2	1100	3.3	81	5.5	1100	19.2	81
Missing ≥3 SFs	1.0	2161	1.7	34	4.1	2161	24.2	34
EmONC readiness								
Fully EmONC ready	1.8	34	4.5	39	12.3	34	24.0	39
Not ready for 1 or 2 SFs	1.2	1865	4.9	196	6.0	1865	18.6	196
Not ready for ≥3 SFs	0.9	1547	3.2	59	3.1	1547	17.9	59
Motor vehicle transport								
No	1.0	2434	2.6	42	4.5	2434	12.3	42
Yes	1.3	1012	4.8	252	5.4	1012	20.3	252
SBAs per 100 deliveries								
< 1	0.5	199	1.6	4	0.9	199	17.6	4
1 to 2	0.7	878	2.2	15	2.1	878	12.8	15
2 to 3	0.9	687	2.8	20	3.4	687	15.3	20
3 to 4	1.1	430	3.9	24	4.8	430	10.5	24
4 to 5	1.5	274	5.7	33	5.7	274	18.5	33
5 to 6	1.6	175	6.4	24	6.9	175	19.0	24
6 to 7	1.7	114	4.5	21	6.7	114	17.7	21
7 to 8	1.4	81	5.8	12	6.7	81	16.9	12
9 to 10	1.5	70	5.5	18	9.0	70	14.6	18
> 10	1.7	429	4.5	118	12.0	429	23.8	118
Annual deliveries								
Lowest quartile (≤ 196 per year)	1.6	826	3.4	64	7.2	826	23.3	64
Lower middle quartile (197–386 per year)	1.1	925	4.2	26	5.3	925	23.6	26
Upper middle quartile (387–651 per year)	0.9	913	3.3	39	3.7	913	17.3	39
Highest quartile (≥652 per year)	0.6	782	5.2	165	2.8	782	17.4	165

We examined the influence of a MWH on the PDR and DOCR while controlling for region, managing authority, location, EmONC status and readiness, availability of transport, density of SBA and annual volume of deliveries. We modeled health centers and hospitals separately and present results in Table 4.

**Table 4:** Regression coefficients on perinatal deaths and direct obstetric complications per 100 deliveries

	Model 1: Regression coefficients on perinatal deaths per 100 deliveries (ln)				Model 2: Regression coefficients on rate of direct complications per 100 deliveries (ln)			
	Health Centers		Hospitals		Health Centers		Hospitals	
	Coef.	*p*-value	Coef.	*p*-value	Coef.	*p*-value	Coef.	*p*-value
Maternity waiting home	**0.133**	< 0.01	**−0.473**	< 0.01	− 0.047	0.363	**− 0.492**	0.011
Region								
Urban (Addis Ababa, Dire Dawa, Harari)	**		**		**		**	
Agrarian (Amhara, Oromia, SNNP, Tigray)	**0.736**	< 0.01	**0.596**	< 0.01	**− 0.773**	< 0.01	0.372	0.107
Pastoralist (Gambella, Benishangul-Gumuz, Afar, Somali)	**1.142**	< 0.01	**0.607**	0.016	**−1.302**	< 0.01	0.401	0.253
Managing authority								
Public	**		**		**		**	
Private, for-profit	0.484	0.069	**−0.366**	0.041	0.000	1.000	**0.602**	0.015
Private, not-for- profit	−0.090	0.629	−0.003	0.989	0.489	0.078	0.205	0.515
Rural	**−0.191**	< 0.01	0.129	0.537	**−0.300**	< 0.01	−0.529	0.071
Number of EmONC signal functions performed	**0.075**	< 0.01	**0.147**	< 0.01	**0.078**	< 0.01	0.045	0.405
Number of EmONC signal functions staffed and equipped	**0.080**	< 0.01	0.016	0.748	**0.180**	0.000	0.090	0.203
Any motor vehicle transport	− 0.022	0.567	**0.515**	< 0.01	**0.112**	0.038	0.339	0.116
SBAs per 100 deliveries	**0.051**	< 0.01	**0.082**	< 0.01	**0.170**	< 0.01	0.047	0.140
Annual deliveries								
lowest quartile (≤ 196 per year)	**		**		**		**	
lower middle quartile (197–386 per year)	**− 0.470**	< 0.01	− 0.063	0.764	**− 0.222**	0.011	0.194	0.515
upper middle quartile (387–651 per year)	**−0.739**	< 0.01	−0.205	0.293	**−0.347**	< 0.01	− 0.278	0.312
highest quartile (≥652 per year)	**−1.189**	< 0.01	0.181	0.337	**−0.641**	< 0.01	−0.208	0.446
Constant	**− 5.641**	< 0.01	**−6.073**	< 0.01	**−4.175**	< 0.01	**−3.851**	< 0.01
	R^2^ = 0.3070		R^2^ = 0.3600		R^2^ = 0.2917		R^2^ = 0.1596	
	Adj R^2^ = .3033		Adj R^2^ = 0.3267		Adj R^2^ = 0.2877		Adj R^2^ = 0.1160	
	Obs = 2442		Obs = 264		Obs = 2344		Obs = 265	

Coefficients significant at *p* < 0.05 are bolded. ** reference category

The reference groups were the same for health centers and hospitals: region – urban; managing authority – public; MWH – none; urban/rural-urban; transport – no vehicle on site; and delivery volume -- low (fewer than 197 deliveries per year). EmONC status, readiness, and SBAs per 100 deliveries were treated as continuous variables.

### Association between the perinatal death rate and facilities with MWHs and other facility characteristics

The average PDR at a health center was e^-5.641^, or 0.35 deaths per 100 deliveries. Holding all other variables constant, a health center with a MWH had a PDR 13% higher, or 0.40 deaths per 100 deliveries, than health centers without a MWH. The average PDR at a hospital was e^-6.073^ or 0.23 deaths per 100 deliveries. However, holding all variables constant, the presence of a MWH predicted a PDR that was 47% lower, or 0.12 deaths per 100 deliveries, than hospitals without a MWH.

Variables that independently predicted a significantly higher PDR among health centers included: location in an agrarian or pastoralist region (between 74 and 114% higher PDRs than similar facilities in urban regions), each additional EmONC signal function performed (8% increase per function), each additional EmONC signal function that the facility was ready to provide (8% increase), and number of SBAs per 100 deliveries (5% increase in PDR for each unit increase in SBA ratio).

Independent predictors of significantly lower PDRs among health centers included rural residence (19% lower) and a larger annual volume of deliveries (47 to 119% lower PDR relative to facilities in the lowest quartile).

Among hospitals, independent predictors of a significantly higher PDR included: location in agrarian or pastoralist regions (60 and 61% increase, respectively), each additional EmONC signal function performed (15% increase per function), presence of a motor vehicle (52% increase), and SBAs per 100 deliveries (8% increase per unit increase).

In addition to the presence of a MWH (with a 47% decrease), the only other significant predictor of lower PDRs among hospitals was private, for-profit status (37% decrease).

### Association between direct obstetric complication rate and facilities with MWHs and other facility characteristics

Among health centers, the average DOCR was e^-4.175^, or 1.5 complications per 100 deliveries. Holding all other variables constant, a health center with a MWH had a direct complication rate that was 4.7% lower than a health center without a MWH, but the difference was not statistically significant. Among hospitals, the average complication rate was e^-3.851^ or 2.1 complications per 100 deliveries. Holding all variables constant, the presence of a MWH significantly predicted a complication rate that was 49% lower, or 1.1 complications per 100 deliveries, than hospitals without a MWH.

Independent variables that significantly predicted a higher DOCR among health centers included the number of EmONC signal functions performed (each additional EmONC signal function increased the complication rate by 7.8%), EmONC signal function readiness (each additional signal function that the facility was ready to provide increased the complication rate by 18%), presence of a motor vehicle (11% increase), and SBAs per 100 deliveries (17% increase for each unit increase).

Significant predictors of lower DOCRs among health centers included location in agrarian or pastoralist region compared to urban regions, rural rather than an urban setting, and a higher volume of deliveries relative to the lowest quartile of facilities. Among hospitals, the only other variable significantly associated with the DOCR was management by a private, for-profit hospital that predicted a 60% increase in the DOCR compared to public facilities.

## Discussion

This was the first national assessment of all MWHs in Ethiopia and was conducted at a time of rapid growth in infrastructure in general and of MWHs in particular. The last 5–10 years saw an expansion of MWHs from less than a dozen sites, located primarily within or alongside hospitals [24], to MWHs in 1943 (56%) health centers and 58 (18%) hospitals in 2016.

Despite the expansion of MWHs, the current distribution points to gaps in the ministry’s desired target of a MWH at every health center. The region of Gambella had no MWHs and Afar and Somali had few. Highly urbanized regions and city administrations also had few MWHs, but utilization of skilled maternity care is high in these locations [11, 18]. The prioritization of targeting rural agrarian or pastoralist regions for MWHs appears to be a sound strategy.

Conditions at MWHs varied widely but tended to be better among MWHs at hospitals than at health centers which might be due to more funds for investment at hospitals. Regional variation in infrastructure and amenities showed MWHs in the pastoralist regions often at a disadvantage, indicating the need for government and communities to improve conditions of existing MWHs, in addition to increasing their numbers.

Because MWHs are considered a potentially effective strategy to reduce maternal and newborn morbidity and mortality [21], the correlations between the presence of a MWH and perinatal mortality and maternal morbidity begin to fill certain knowledge gaps. We recognize that the two outcomes – the perinatal mortality rate and the direct obstetric complication rate – may not be conceptually equivalent. High perinatal mortality is unequivocally undesirable. A high DOCR is less straight forward since not all serious obstetric complications are preventable. Observational data from catchment areas where MWHs have existed for years and are well accepted by communities have demonstrated a decline in facility-based obstetric complication rates [24]. This is highly dependent on a facility’s ability to provide emergency care and echoes the MWH Cochrane Review statement that facilities with MWHs should provide emergency obstetric care [21]. In Ethiopia, two studies compared newborn and maternal outcomes at hospitals with MWHs with outcomes of women who were admitted directly to the hospital [25, 26]. Braat et al. [26] were able to extend their comparison to a second but similar hospital that had no MWH. Their findings help put into perspective what we might expect from our observational study.

Theoretically, MWHs should play a protective role if occupants are closely monitored for fetal and maternal well-being, and if at the first sign of danger, they can be transferred to a special care ward within the hospital or from the health center to a hospital. In the studies by Kelly et al. and Braat et al. [25, 26], MWH occupants were less likely to die, had fewer stillbirths, fewer uterine ruptures, and higher rates of cesarean sections than women who were admitted directly to the hospitals. Our study, which used a very different design and sample, had mixed findings: at hospitals, the presence of a MWH indicated a 47% lower perinatal mortality as well as a 49% lower obstetric complication rate compared to hospitals without a MWH. At health centers, on the other hand, presence of a MWH indicated a significantly higher PDR (13% higher) but had no effect on the DOCR, compared to facilities without a MWH.

The mixed results found between health centers and hospitals, and between the PDR and the DOCR may in part be explained by limitations of the data and the type of patients received where hospitals are likely to receive more women with obstetric complications than health centers. On the other hand, our study design restricted us to cross-sectional data for some variables and retrospective aggregated service statistics for our outcomes, both of which are likely to be undercounted. Rather than examining the perinatal mortality rate, we would have preferred to examine intrapartum stillbirths and very early neonatal deaths and exclude the macerated stillbirths since these occur before intrapartum care is provided. The health information system did not distinguish between macerated and intrapartum stillbirths. The aggregated facility statistics did not allow us to identify which women stayed at the MWH; thus, our focus on the facility level outcome (PDR or DOCR) was a departure from most studies that attempt to assess the effectiveness of MWHs at the individual patient level. Given the rapid acceleration of MWHs, it is possible that some of the MWHs were established in 2015, the year for which the statistics were gathered, and therefore, it is possible that women were misclassified since not all women had the option of using the MWH.

## Conclusions

The impact of MWHs on maternal and perinatal outcomes appears promising and as MWHs continue to expand, so should the efforts of service providers to regularly monitor (and refine) these outcomes to document their impact. More focus should be given to establishing MWHs in hospitals where their availability potentially means preventing a larger number of perinatal deaths and direct obstetric complications as compared to health centers. The findings are applicable to informing regional maternal and newborn improvement strategies, indicating gaps in the distribution and conditions of the MWHs outlined in national and global guidelines. Policymakers and program planners need to pay close attention to the expansion and furnishing of MWHs in pastoralist regions.

Further investigation is warranted to understand why perinatal mortality was higher in health centers with MWHs than in those without. Moreover, a well-designed trial is needed to examine the effects of MWHs on the critical outcomes including maternal mortality and severe morbidity.

## Data Availability

The data belong to the Ethiopia Federal Ministry of Health and the EPHI. Requests for permission to access the data should be addressed to EPHI.

## Abbreviations

AMDD: Averting Maternal Death and Disability Program
ANC: Antenatal Care
BEmONC: Basic Emergency Obstetric and Newborn Care
CEmONC: Comprehensive Emergency Obstetric and Newborn Care
DOCR: Direct Obstetric Complication Rate
EmONC: Emergency Obstetric and Newborn Care
EPHI: Ethiopian Public Health Institute
HSTP: Health Sector Transformation Plan
LMICs: Low- and Middle-Income Countries
MCH: Maternal and Child Health
MWH: Maternity Waiting Homes
PDR: Perinatal Death Rate
PHCU: Primary Health Care Unit
SBA: Skilled Birth Attendants
SNNP: Southern Nations, Nationalities, and People’s Region

## Footnotes

1 A maternity waiting home is a room within or a free-standing structure close to a hospital or health center that provides basic or comprehensive emergency obstetric care. Expectant women who live in remote areas and have limited access to care locally can stay at a MWH towards the end of their pregnancy, usually in the last two or three weeks of pregnancy. The aim of the MWH is to improve accessibility to skilled care and reduce maternal and neonatal morbidity and mortality.
